# Super-Resolution Ultrasound Imaging Scheme Based on a Symmetric Series Convolutional Neural Network

**DOI:** 10.3390/s22083076

**Published:** 2022-04-16

**Authors:** Lakpa Dorje Tamang, Byung-Wook Kim

**Affiliations:** Department of Information and Communication Engineering, Changwon National University, Changwon 51140, Korea; ld.tamang25@gmail.com

**Keywords:** ultrasound imaging, image enhancement, super resolution, convolutional neural network, deep learning

## Abstract

In this paper, we propose a symmetric series convolutional neural network (SS-CNN), which is a novel deep convolutional neural network (DCNN)-based super-resolution (SR) technique for ultrasound medical imaging. The proposed model comprises two parts: a feature extraction network (FEN) and an up-sampling layer. In the FEN, the low-resolution (LR) counterpart of the ultrasound image passes through a symmetric series of two different DCNNs. The low-level feature maps obtained from the subsequent layers of both DCNNs are concatenated in a feed forward manner, aiding in robust feature extraction to ensure high reconstruction quality. Subsequently, the final concatenated features serve as an input map to the latter 2D convolutional layers, where the textural information of the input image is connected via skip connections. The second part of the proposed model is a sub-pixel convolutional (SPC) layer, which up-samples the output of the FEN by multiplying it with a multi-dimensional kernel followed by a periodic shuffling operation to reconstruct a high-quality SR ultrasound image. We validate the performance of the SS-CNN with publicly available ultrasound image datasets. Experimental results show that the proposed model achieves a high-quality reconstruction of the ultrasound image over the conventional methods in terms of peak signal-to-noise ratio (PSNR) and structural similarity index (SSIM), while providing compelling SR reconstruction time.

## 1. Introduction

Image super resolution (SR) [1,2] refers to the reconstruction of a high-resolution (HR) image from its counterpart in the low-resolution (LR) space. Recently, SR image reconstruction has been a prolific area of research in the fields of digital image processing and computer vision because of its ability to solve the inherent resolution limitation problems of low-cost image sensors. Real-world applications for SR range from enhancement of blurred and noisy images/videos into high-definition (HD) images/videos [3], robust pattern recognition [4], and microscopic object detection [5]. In addition, a higher-quality image obtained from SR leads to a higher degree of accuracy in medical imaging analysis, where proper and accurate localization of tumors is required. Ultrasound images cannot always be acquired with ideal image quality due to the phenomena in the transmission medium and intrinsic properties of ultrasound, especially at low spatial resolution. Although the use of high-quality medical imaging equipment is widely practiced, ultrasound images might have various image artifacts, such as blurring phenomenon caused by the propagation of ultrasonic waves, and noise due to the ultrasound beam characteristics [5]. These kinds of artefacts in medical images might lead to poor visual quality of textural, and spatial components. Moreover, this could impose a limitation on posterior analysis of medical conditions, possibly leading to false suppositions. In this regard, the requirement for better spatial resolution of the ultrasound images with high textural information of lesions and blood vessels is one of the most research topics in the field of medical imaging in recent years. Within the medical imaging domain, ultrasound is a versatile and widely practiced diagnostic tool. Compared to other medical imaging modalities, ultrasound usually produces poor spatial resolution of deep tissues due to the wavelength-dependent inverse relation between penetration depth and resolution [6]. Ultrasound imaging has a resolution constraint by diffraction due to the scale of its wavelength and relies on the echo of deep tissue, making it difficult to reconstruct high quality images due to the tissue variability and compressibility [7]. In ultrasound medical imaging, the presence or absence of a lesion is determined by observing the shape of the region of interest, the degree of blood flow and the opposing smoothness. In such a scenario, the role of textural information of such lesions in the spatial component of the ultrasound image is vital for the proper diagnostic treatment. Ultrasound images with visible, vivid textural patterns, and high spatial resolution are therefore essential for the accuracy of medical diagnosis. The exploitation of the SR task to effectively increase the resolution of poor-quality ultrasound images has recently prompted extensive interest of research [8,9].

In previous research, multiple SR studies based on interpolation techniques [10,11], and sparse representation [12,13] have been explored for enhancing the quality of images to the desired resolution. Moreover, in [14], to improve the temporal resolution of the ultrasound imaging, an imaging sequence and signal processing approach was proposed by employing deconvolution and data acquisition based on spatio-temporal interframe-correlation. Recently, due to the paradigm shift in image processing technology, the deep learning (DL) framework has been considered for SR tasks [15,16,17,18] in various applications. These DL techniques [19,20] offer the significant prospect of providing better SR quality than conventional schemes because of their ability to learn non-linear feature representatives from image data. Popular frameworks, such as ResNet [21], DenseNet [22], and recurrent neural networks (RNNs) [23], have been utilized in SR tasks with natural images. In the medical imaging domain, multiple studies [5,24,25,26] have investigated DL-based SR approaches. Especially for ultrasound imaging, DL technology has been incorporated to improve the quality of ultrasound imaging in studies such as [27,28,29]. Liu et al. [27] proposed a perception consistency ultrasound image SR technique based on a self-supervised cycle generative adversarial network (CycleGAN) framework. This study integrated multi-level feature loss and the adversarial characteristics of the generator during the SR task to balance the visual similarity between real data and reconstructed data. In [28], a multi-frame SR approach was proposed for ultrasound images from a set of LR images. To cope with motion estimation while performing multi-frame SR, this research proposed a DL network that obtains HR images by reducing the effect of existing noise in LR ultrasound images. Similarly, in [29], a fast medical image super-resolution (FMISR) method was proposed where a set of three hidden layers with a mini-network in between are used for complete feature extraction. This is followed by a sub-pixel convolution (SPC) layer for successful image up-sampling. Although these techniques yielded convincing results in terms of image quality, they do not exploit the low-level features of the input image throughout the network. The spatial feature of any image is highly constrained within the low-level features extracted from the initial layers of the CNN. The need for low-level features while implementing any SR task is therefore important, as it can provide additional information for reconstructing the high-frequency details of the HR image [30]. In addition, when low-level features are passed into the latter layers through skip connections, the vanishing gradient problem can be alleviated. Furthermore, conventional techniques do not take feature redundancy into account, which instigates the risk of learning the same feature multiple times, resulting in limited reconstruction quality of the SR imaging.

To overcome these shortcomings, we propose a novel SR model for ultrasound imaging, referred to as a symmetric series convolutional neural network (SS-CNN). The proposed model comprises two parts: a feature extraction network (FEN) and up-sampling. To extract vital features that contain fine structural details of ultrasound images, a FEN consisting of a symmetric series of two feed forward convolutional neural networks (CNNs) was designed. Subsequent layers in the SS-CNN are concatenated with skip connections to utilize low-level features of the LR input image and to minimize the vanishing gradient problem. This leads to the reconstruction of rich high-frequency details from the input image while conducting the SR task. In addition, it provides the benefits of the compact network structure with minimal feature redundancy by allowing feature reuse between symmetric convolutional series. In doing so, a considerable number of features, including details of high-frequency components, will be propagated to the final layer of the FEN. Then, the up-sampling procedure is conducted by employing the SPC layer as an up-sampling operator. In this layer, the feature map generated by the FEN is multiplied by a multi-dimensional kernel and then subjected to a periodic shuffling operation to finally generate the output HR image. In the test results obtained using a publicly available ultrasound image database, we observed that the proposed scheme offered superior performance in terms of peak signal-to-noise ratio (PSNR) and structural similarity index (SSIM) and a fair SR reconstruction speed, compared to state-of-the-art SR approaches.

The rest of the paper is organized as follows. In Section 2, the proposed system is discussed. Experiments and results are discussed in Section 3. Finally, in Section 4, conclusions are reported.

## 2. The Proposed System

**Figure 1:**
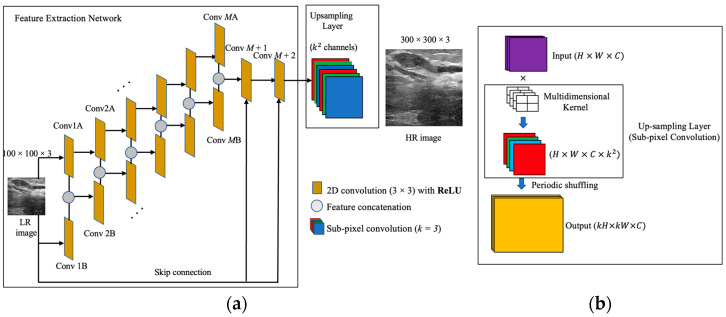
Schematic representation of the SS-CNN model. (**a**) Feature extraction network, and (**b**) Up-sampling layer.

The system architecture of the proposed scheme is depicted in Figure 1. The network is comprised of the FEN and an up-sampling layer. The FEN is responsible for extracting imminent features from the input image and forwarding them to the up-sampling layer. The up-sampling layer then enhances image resolution by upscaling the output feature map from the preceding layer to produce the HR output.

### 2.1. The Feature Extraction Network

Feature extraction in SR tasks can be formulated as a sampling problem where perfect reconstruction of the desired features is required. The main purpose of this network is to extract image blocks from LR images. The dimensions of the extracted blocks remain in the LR space. This network basically extracts local hierarchical features of an input LR image in different stages, from shallow to deep. To acquire a more hierarchical representations of the input image, increasing the depth of the FEN is the general approach in the DL paradigm. The deep layers can prominently extract various ranges of useful feature representations (low to high). However, the huge gradient loss from the backpropagation process must be dealt with. Therefore, an alternative structure for building a FEN would be passing the same input to a series of two symmetric convolutional layers, where the feature maps of the corresponding layers are concatenated. With the symmetric series, abundant semantic information of the LR input can be explored, from which better perceptual quality of the SR output can be achieved. Note that the resolution of ultrasound images is impeded by the fundamental limits of diffraction, creating a long-standing trade-off between resolution and penetration. Accurate image reconstruction is therefore crucial for the SR task and can be achieved by using a robust feature extractor prior to up-sampling.

In the SS-CNN model, the FEN consists of a symmetric series of two convolutional layers: Conv A and Conv B. Each series holds M convolutional layers (M being the depth of each series) followed by a rectified linear unit (ReLU) as a non-linear activation function. The input for the network is the LR samples created by down-sampling the original HR samples. During forward propagation, the feature maps generated by subsequent Conv A and Conv B layers are concatenated. This allows the FEN to learn global features of the ultrasound images by concealing together the local features of both inputs from each subsequent layer. In this way, the network avoids learning redundant features and instead learns a unique representation of the input produced by each layer. The network also facilitates sufficient regularization as the weights are shared between each symmetric convolutional layer, so any external regularization techniques are not required. Besides, skip connections are introduced between the input image and the latter convolutional layers of the FEN. Good SR performance is guaranteed when the neural network has the collective knowledge of multiple levels of feature information [30,31]. In particular, low-level features can potentially provide additional information needed when reconstructing high-frequency details of an image. Skip connections not only help preserve spatial information, but also create short paths for gradients to flow through the network, allowing networks to be trained without gradient vanishing and overfitting problems. Therefore, skip connections allow image features to be preserved from the lower-level layers, and bypass the important textural details to high level layers in the CNN, resulting in high quality HR images in the SR process.

The detailed structure of the proposed FEN with M = 6 is presented in Table 1. The convolutional layers of both series use 64 filters and 3 × 3 kernel sizes to convolve over an image during single convolution. Small-sized filters are preferred because they facilitate easier weight sharing and reduce the network complexity. The cumulative features extracted by concatenating simultaneous feature maps from both series are forwarded to two convolutional layers at the latter stages of feature extraction. The later layers use thirty-two and three filters, respectively, with a receptive size of 1 × 1. To achieve potential improvement in SR reconstruction performance, both later layers are concatenated with the input image via skip connections. This ensures that the final layer of the feature extraction network contains enough textural information from the input image.

Note that the design of this network focuses on extracting global features from the input image. Therefore, to achieve this, the convolutional layers from both series have an identical size for the receptive field so the concatenated feature maps from the subsequent layers of Conv A and Conv B accommodate sufficient image information. In addition, to preserve important details of the feature maps produced, pooling and down-sampling layers are not used throughout the network.

### 2.2. Up-Sampling Layer

The up-sampling layer is responsible for reconstructing a high-quality HR image from the image features extracted from the FEN. Generally, in conventional DL-based SR tasks, a transposed convolution (deconvolution) layer is used for up-sampling to reconstruct high-quality images [15]. However, since the FEN of the proposed model does not utilize any down-sampling layers (e.g., maxpooling), the transposed convolution layers are not suitable candidates for an up-sampling operation. Moreover, using the deconvolutional layer for up-sampling has the disadvantage of significantly increasing the computational cost and model complexity due to the large numbers of feature maps being fed into it. Therefore, to up-sample the features of an LR image into the high-quality HR space, we used the SPC layer, which is a learnable layer that first multiplies its input with a multi-dimensional kernel to generate multiple feature maps for up-sampling. The multi-dimensional kernel consists of an array of several image upscaling filters, and a large number of same sized feature maps are obtained by performing a convolution operation in the SPC layer. These multiple feature maps are then rearranged or shuffled to generate output with a significantly higher resolution in such a way by multiplying the height and width of the output tensor with upscaling factor. With this, the network can learn to use multiple channels of the LR image features obtained from the FEN to represent a single HR image. Unlike the deconvolutional layer, which explicitly enlarges the feature map to increase resolution, the SPC layer expands the number of feature maps and applies a specific region-mapping criterion to obtain the HR output. From this, there is uniformity in the resolution of the feature maps obtained from the FEN, and the model becomes computationally less expensive.

In the SPC layer, the sets of weights for the convolutional kernel are independent from each other during convolution. For each feature map, this layer can generate $k^{2}$ channels in a single upscaling, where k is the upscale factor. Therefore, by choosing the desired value of k, the SPC layer can map the image to the HR space from its LR counterpart in a single upscaling. From Figure 1b, we can clearly see that by convolving the input of dimensions H×W×C (height, width, and channels, respectively) with a multi-dimensional kernel, we can obtain output with the same dimensions as the input but with additional $k^{2}$ channels. Subsequently, a periodic shuffling (PS) operation is used to reshape the output channels to the desired HR output. This operation rearranges a tensor with dimensions ($H\times W\times C\;.\;k^{2}$) into a (kH×kW×C) matrix. From this, $k^{2}$ channels are distributed to the spatial dimension of the image, where both height and width are multiplied by k to generate the HR output. Throughout this process, the up-sampling layer is able to generate HR output with a single upscaling of the feature maps obtained from the FEN without using a complex and computationally expensive deconvolution operation.

### 2.3. Datasets

**Figure 2:**
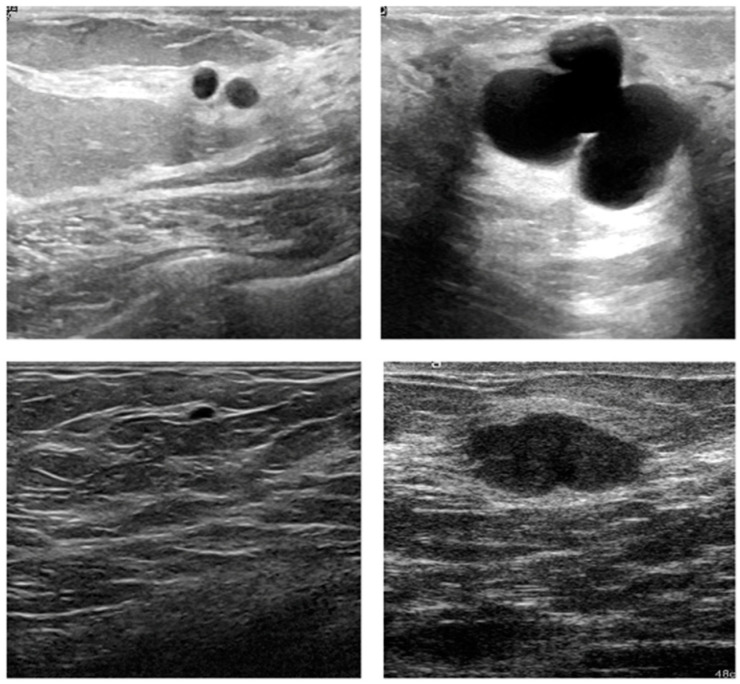
Image samples for training: top row, from Dataset A, and bottom row, from Dataset B.

The proposed model was evaluated using two publicly available ultrasound image datasets, with examples as shown in Figure 2. The breast ultrasound images (BUSI) dataset [32] (Dataset A) was collected in 2018 at Baheya Hospital, Cairo, Egypt, for early detection and treatment of women’s cancer. A total of 780 images with an average resolution of 500 × 500 pixels were acquired from female candidates between ages 25 and 75 years. The dataset consists of 133 normal images (without cancer), 437 images with cancer, and 210 images with benign tumors. The whole dataset was divided into three parts: training with 521 images, validation using 109 images, and testing 150 images. The other dataset [33] (Dataset B) was collected from the UDIAT Diagnostic Center of the Parc Tauli Corporation in Sabadell, Spain. This dataset was collected in 2012 using an 8.5 MHz Siemens ACUSON Sequoia C512 HD linear array transducer. The dataset contains a total of 163 images with a mean resolution of 760 × 570 pixels, where 53 are cancer images, and the remaining 110 are images of benign lesions. The main purpose for creating this dataset was detecting the lesions. However, in this research, we employed it for the SR task. Out of the entire dataset, 110 images were selected for training and subjected to data augmentation later, while the remaining 30 and 23 images were used for validation and testing, respectively.

### 2.4. Data Augmentation

The images available from the datasets were not numerous enough to prevent overfitting and for properly training various weights of the SS-CNN structure. Therefore, we expanded the dataset’s limited availability by adapting a data augmentation technique. In the experiment, various augmentation techniques, such as rescaling the original pixel values between [0, 1], 10° image rotation, horizontal and vertical shifting of 2 pixels, horizontal flipping, and random zooming operation, were utilized. During data augmentation, we increased the dataset sizes by adding synthetic instances to the existing training set. These instances were created by applying domain-specific techniques, such as geometric transformations, to the original samples. Dataset A and Dataset B were augmented to obtain 880 and 862 images for training, respectively.

### 2.5. Training

**Figure 3:**
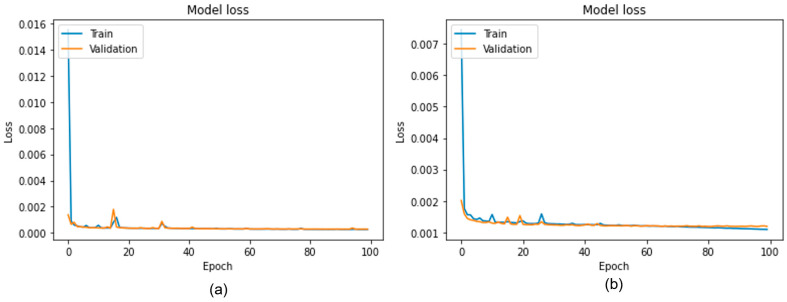
Learning curves from training on the validation datasets: (**a**) dataset A, and (**b**) dataset B.

For the SR task, the training process follows a self-supervised learning strategy in which manual labelling of the training data is not required. The LR images are first generated by down-sampling their HR counterparts from the training sets. The output generated during forward propagation was compared with the original HR images to compute the loss function. The parameters used while training the network are presented in Table 2. The whole network was optimized by minimizing the mean square error (MSE) loss between the model’s prediction and its discrete HR ground truth images. For SR imaging, the previous study in [34] showed robust image quality with the adaptive momentum (Adam) optimizer. Therefore, to handle sparse gradients due to the noisy spatial resolution of LR images, we used the Adam optimizer with a learning rate of 0.001. During training, the model learns at the level of individual image pixels. To cope with the input image dimensions for the designed network, the images were resized to 300 × 300 pixels. For training and validation purposes, 80% and 20% of the dataset images were respectively allocated. The whole model was trained within 100 epochs with a smaller batch size of 8 to improve generalization of the proposed model. Figure 3a,b show the learning curves of the SR model with datasets A and B, respectively, for both training and validation datasets. In both scenarios, the training and validation loss converge with respect to epochs. No sign of overfitting can be observed, which demonstrates effective training of the model up to the point where saturation in training loss and accuracy was achieved.

**Figure 4:**
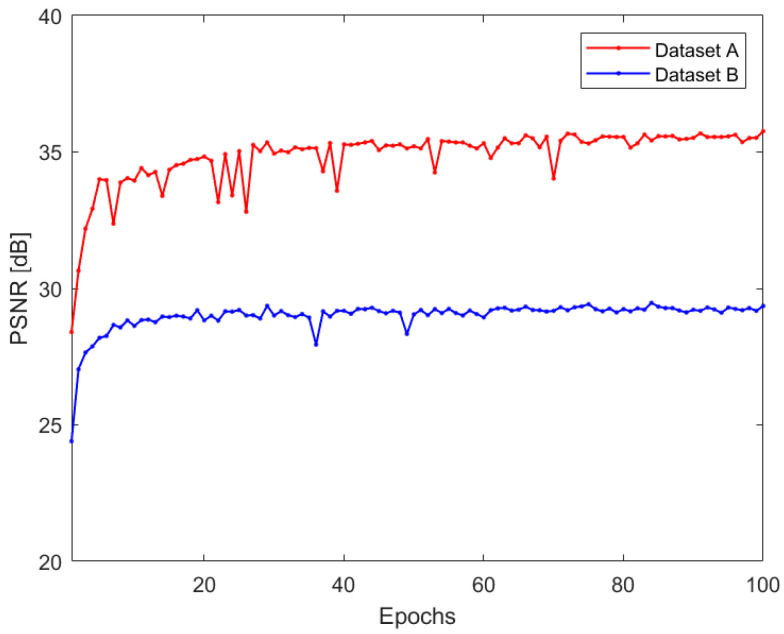
PSNR vs epochs of the SS-CNN model from the different datasets.

**Figure 5:**
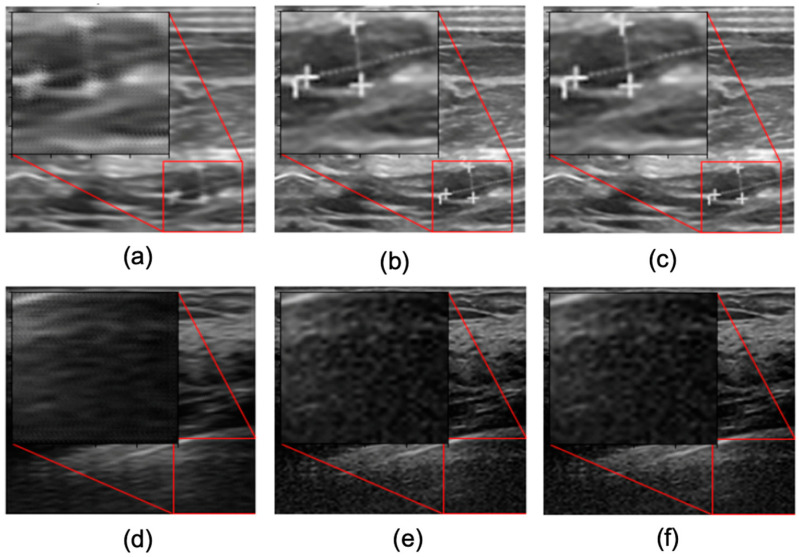
Reconstructed images at the beginning and the end of the training phase: (**a**) Dataset A: HR at epoch 1 (28.40 dB), (**b**) Dataset A: HR at epoch 100 (35.76 dB), (**c**) Dataset A: Ground truth, (**d**) Dataset B: HR at epoch 1 (24.40 dB), (**e**) Dataset B: HR at epoch 100 (29.36 dB), and (**f**) Dataset B: Ground truth.

Furthermore, as the SR model learns to enhance the visual quality of the LR image upon training, therefore, PSNR increments relative to the training epochs are shown in Figure 4. This figure represents how the model is learning to enhance the visual quality of the LR images while the training continues. For Dataset A, the PSNR increases from 28 dB and finally reaches 35 dB. Here, we can observe saturation of PSNR after 70 epochs. In contrast, for Dataset B, the PSNR during the initial stage of training was 24 dB, which finally reached 29 dB during the final epochs. This indicates that loss in the model converges with the training epochs, and therefore, the model was trained properly on both datasets. Figure 5 shows the reconstructed images at the beginning and the end of the training phase. As shown in Figure 5b–d, the reconstructed image presents an enhanced image quality and valuable details. A significant difference in the visual quality of the image can be observed between the first and final epochs. Thus, we can consider the model we proposed to have learned the given datasets well.

## 3. Experiments and Results

This section addresses the performance evaluation of the proposed model by analyzing reconstructed SR images from publicly available ultrasound imaging datasets. The whole experiment was performed on an AMD Ryzen processor at 3.7 GHz with 32 GB of RAM and an RTX 2080 Ti GPU running Windows 10 and using Keras 2.3.1 with a TensorFlow 1.15 library in a Python (version 3.6) environment. This section presents a detailed discussion regarding the comparison between the proposed SR model and existing state-of-the-art approaches with respect to image quality and reconstruction speed.

### 3.1. Visual Assessment

The visual quality of the SR images was assessed based on image distortion and noise level during reconstruction. In this regard, PSNR and SSIM were used for comparing HR image restoration results. The former measures the ratio between the maximum possible value of the signal and the power of the noise that affects the quality of its representation. The latter is based on factors, such as luminance, contrast, and structure, that are appropriate for the human visual system. For the performance comparison, we evaluated the proposed SS-CNN model, bicubic interpolation, and two other state-of-the-art methods: ESPCN [17], and FMISR [29]. The bicubic interpolation technique is one of the most common and fundamental techniques for sharpening and enlarging images in the image processing paradigm which attempts to fit a surface between four corner points of a pixel grid. In this technique, the desired value of a pixel is estimated within the 4 × 4-pixel grid, i.e., 16 pixels of an image by performing interpolation within the grid. For the up-sampling procedure after feature extraction, the ESPCN SR model also utilizes an SPC technique. Note that we evaluated these models on top of publicly available ultrasound image datasets [33,34]. Thus, the reported values might slightly deviate from those presented in the original paper, which were obtained from different datasets.

**Figure 6:**
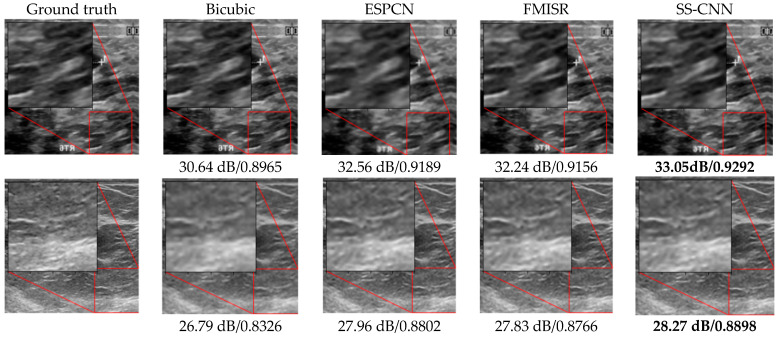
Ground truth HR image and reconstructed HR images generated from various reference methods including the proposed model. PSNR and SSIM values are shown below each sub-figure. Top row (Dataset A), and bottom row (Dataset B). Bold characters represent the highest value of PSNR and SSIM.

Figure 6 presents a visual quality assessment of the HR images reconstructed using the bicubic method, two state-of-the-art DL-based SR methods (ESPCN [17], and FMISR [18]), and the proposed SS-CNN model. We can see that the bicubic method exhibits the worst performance, where an excessive number of visual artifacts, such as blurriness and image noise, can be observed. The HR images reconstructed with ESPCN [17] and FMISR [29] endured comparatively fewer visual artifacts than the bicubic method. However, their feature extraction capability was limited, and thus, these techniques failed to preserve the high-frequency details of the images. On the other hand, the high frequency components represented by the sharp edges in the image are well preserved in the SS-CNN based SR reconstruction model when compared to the adversary techniques. We can see that, of all the other methods, the SS-CNN model provided the best visual quality in the reconstructed HR images. In particular, the spatial resolution of the tumor tissues in the cancerous breast imagery is visually enhanced by using SS-CNN model as shown in the red square box in Figure 6. This was validated by the PSNR and SSIM values, which are significantly higher than those provided by the reference schemes. The visual perception and textural patterns of the obtained HR ultrasound images avoided large distortions when compared to the input LR images. These convincing results can be attributable to the symmetric network structure of the FEN, with subsequent feature concatenations to extract significant spatial features from the pixel space. The concatenation of subsequent features from each layer of the symmetric convolutional network series allowed preserving the vital spatial and textural information from the input image, thereby yielding less blurry HR images with relatively minimal visual artifacts. Moreover, the combination of features from the input image with high-level features during the latter stage of the feature extraction network via skip connections provided complementary information from the original image to enhance the HR output.

Table 3 summarizes the quantitative results where the proposed SS-CNN model is compared with state-of-the-art DL-based SR models. For performance comparisons, the image quality of the reconstructed HR image and the time consumed for HR image reconstruction were assessed. Mean values for PSNR and SSIM were recorded for all the test images, which were unseen during training. For SR testing time, we recorded the total time taken for the LR image to be converted into its corresponding HR counterpart. It can be observed that for both datasets, PSNR and SSIM provided by the SS-CNN outperformed all the reference algorithms. In particular, the SS-CNN model achieved a PSNR improvement of 2.3, 0.61, and 0.88 dB, respectively, from bicubic interpolation, and ESPCN and FMISR state-of-the-art SR techniques with Dataset A. With Dataset B, the SS-CNN model improved the PSNR by 1.48, 0.17, and 0.62 dB, respectively. Similarly, the SSIM value was incremented by 0.0317, 0.0154, and 0.0167 with Dataset A and by 0.0325, 0.001, and 0.0031 with Dataset B, when compared to bicubic interpolation, ESPCN, and FMISR, respectively. As mentioned, the significant PSNR gain was achieved due to the symmetric series-based feature extraction network that retained the robust features necessary for up-sampling from the spatial regions of the input LR image.

Note that improving the reconstruction time in the SR task is as important as increasing the quality of the reconstruction. We can see from Table 3 that the SS-CNN not only achieved an impressive PSNR but also a fair SR testing time. All models were tested in identical hardware environments, where a pre-trained model was evaluated on test images of individual datasets, and their mean reconstruction times were recorded. For both datasets, the FMISR technique observed relatively swift reconstruction time. Nevertheless, the proposed method is considered the best choice because it provided overwhelming SR image quality and a satisfactory SR image acquisition time of only 0.310 and 0.296 s for test datasets A and B, respectively.

### 3.2. SR Performance by Varying the FEN Structure

**Figure 7:**
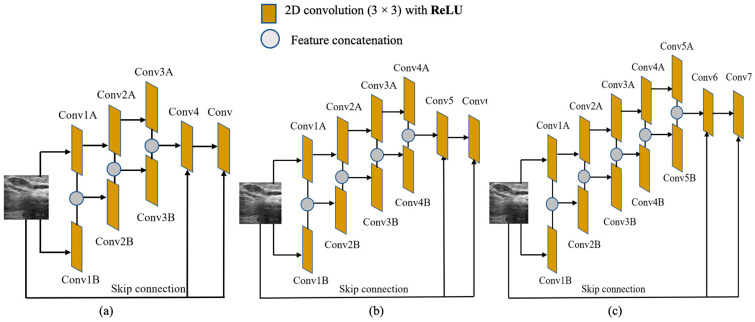
FEN with varying depth. (**a**) M=3, (**b**) M=4, and (**c**) M=5.

In this section, we analyze the performance of the SS-CNN from modifying the network structure of the FEN. For this, the network depths of both convolutional series in the FEN were varied, where multiple candidate depths in the range M∈ [3, 5] were considered. Figure 7a–c show the modified structure of the FEN, where the depth of the symmetric convolutional series, M, gradually increased from 3 to 5. To observe the effect of the depth of the convolutional series, the up-sampling layers remained intact with no structural changes. The total number of filters and their corresponding sizes were also unchanged. As the size of the network was reduced, the total number of trainable parameters was significantly reduced, which in turn decreased the training and testing times. The presented FEN with varying depths was trained with similar experimental settings, as described in Section 2.5, where MSE and the Adam optimizer were used as the loss function and the optimization technique, respectively.

**Figure 8:**
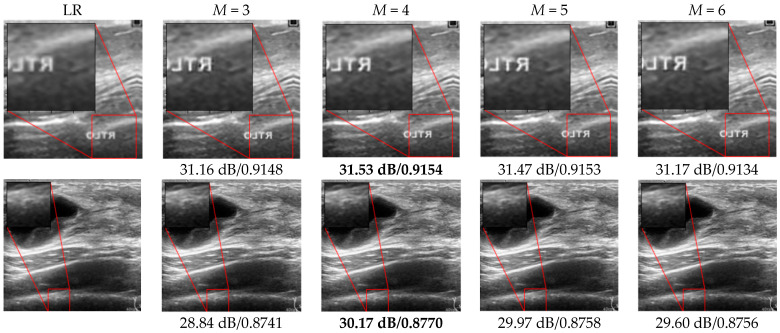
SR results produced by the FEN with variable network depths for Dataset A (top row), and Dataset B (bottom row). PSNR and SSIM values are shown below each sub-figure. Top row (Dataset A), and bottom row (Dataset B). Bold numbers represent the highest values of PSNR and SSIM.

**Figure 9:**
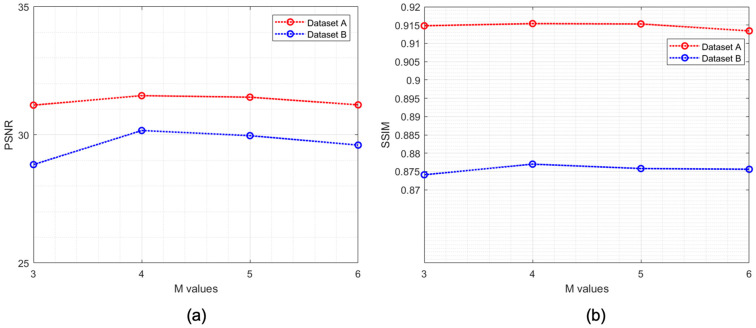
Graphical representation of the (**a**) PSNR [dB] and (**b**) SSIM with respect to the value of *M*.

Figure 8 demonstrates the reconstruction result for SR images when the network depth changed. To measure the effect of noise distortion while varying the size of *M*, PSNR, which is the ratio of the maximum power of a signal to that of noise, was used. In addition, SSIM was used to analyze the visual similarity between the LR and reconstructed HR images. When M is 3 and 6, it can be seen that the image quality is deteriorated as the value of PSNR and SSIM are comparatively lower. Among the various candidates, the best PSNR and SSIM performance was observed in the FEN with M=4. This is also graphically represented in Figure 9, where we can see the PSNR and SSIM for both datasets are highest when the value of *M* is 4. In general DL theory, higher performance from the CNNs can be attributed to the depth of the network. However, this is achieved at the cost of excessive computations and increased model complexity. Unlike this, in our study, feature map concatenation between the convolutional layers of the two symmetric series can provide sufficient feature propagation during the feed forward process. In this regard, with M=4, the proposed model can learn the abstract representation of the input image sufficiently well to yield HR output. On the other hand, upon increasing the depth of the FEN, we can observe degradation in the SR reconstruction performance. This can be credited to the fact that, beyond the saturation point at M=4, the network tends to learn redundant features due to increments in the total number of parameters in the deep model, and eventually, the quality of the reconstructed image is negatively affected. Therefore, a deeper FEN does not always guarantee good performance in SR ultrasound imaging as it introduces the risk of learning redundant feature maps multiple times.

For a quantitative analysis of the FEN with varying M, mean values of the PSNR, SSIM, and SR testing time were measured. We can see from Table 4 that, for both datasets, the PSNR and SSIM recorded their highest levels when M=4. Although shallow networks with a smaller M achieved relatively shorter SR testing times, there is a trade-off in image quality. Note that the optimal M is greatly influenced by the training data and the size of the input and output images. According to the requirements of image reconstruction time and complexity for the objective of SR usage, the depth of the network for an effective FEN design must be determined.

## 4. Conclusions

This paper explored a novel DCNN model with a symmetric series structure for SR reconstruction of ultrasound images. The proposed model comprised two parts: a FEN and an up-sampling layer (for feature extraction and an up-sampling operation, respectively). The subsequent layers of the FEN were concatenated with skip connections to utilize enrichment of multi-scale deep features. The low-level feature maps obtained from both subsequent layers in the DCNN series were concatenated in a feed forward manner to support robust feature extraction and ensure high reconstruction quality in the HR output. The extracted feature maps were passed to the SPC layer, which up-sampled them by multiplying with a multi-dimensional kernel followed by a periodic shuffling operation to generate HR output. Simulation results showed that the proposed scheme outperformed benchmark SR models in terms of image quality, PSNR, and SSIM and achieved a fair SR reconstruction speed. In addition, we demonstrated the influence of the FEN depth on SR reconstruction performance with ultrasound images and proved that a FEN with a depth of 4 achieved prominent PSNR and SSIM values with the given datasets. Simulation results verified both the validity and the efficiency of the proposed DCNN-based SR model. Thus, we are convinced that the high-quality images generated by our SR model can be helpful for further research concerning tumors and tissue structures in ultrasound medical imaging.

## Figures and Tables

**Table 1:** Network Details.

Layers	Input	Filters	*k*	Kernel Size	Output
Conv1A	(100, 100, 3)	64	-	3 × 3	(100, 100, 32)
Conv1B	(100, 100, 3)	64	-	3 × 3	(100, 100, 32)
Conv2A	Conv1A	64	-	3 × 3	(100, 100, 32)
Conv2B	Concat (Conv 1A, Conv1B)	64	-	3 × 3	(100, 100, 32)
Conv3A	Conv2A	64	-	3 × 3	(100, 100, 32)
Conv3B	Concat (Conv 2A, Conv2B)	64	-	3 × 3	(100, 100, 32)
Conv4A	Conv3A	64	-	3 × 3	(100, 100, 32)
Conv4B	Concat (Conv 3A, Conv3B)	64	-	3 × 3	(100, 100, 32)
Conv5A	Conv4A	64	-	3 × 3	(100, 100, 32)
Conv5B	Concat (Conv 4A, Conv4B)	64	-	3 × 3	(100, 100, 32)
Conv6A	Conv5A	64	-	3 × 3	(100, 100, 32)
Conv6B	Concat (Conv 5A, Conv5B)	64	-	3 × 3	(100, 100, 32)
Conv7	Concat (Conv 6A, Conv6B)	32	-	1 × 1	(100, 100, 16)
Conv8	Conv 7	3	-	1 × 1	(100, 100, 3)
Up-sampling layer	Conv 8	-	3	-	(100, 100, 3 × 3^2^)
Periodic shuffling	(100, 100, 3×3^2^)	-	-	-	(300, 300, 3)

**Table 2:** Network Parameters.

Parameter	Value
Loss function	Mean squared error (MSE)
Optimizer	Adam
Learning rate	0.001
Training epochs	100

**Table 3:** Quantitative Analysis of the Proposed SR Model with Current State-of-Art Algorithms. (k=3). Bold numbers represent the highest value of SSIM and PSNR.

Input image	Methods	Mean PSNR [dB]	Mean SSIM	SR Testing Time
Dataset A [32]	Bicubic	31.14	0.8861	-
	ESPCN [17]	32.83	0.9024	0.326 s
	FMISR [29]	32.56	0.9011	0.255 s
	SS-CNN	**33.44**	**0.9178**	0.310 s
Dataset B [33]	Bicubic	30.31	0.8328	-
	ESPCN [17]	31.62	0.8643	0.332 s
	FMISR [29]	31.17	0.8622	0.229 s
	SS-CNN	**31.79**	**0.8653**	0.296 s

**Table 4:** Quantitative Analysis of the Proposed SR Model by Varying the Value of *M*. Bold numbers represent the highest value of SSIM and PSNR.

Dataset	*M*	Mean PSNR [dB]	Mean SSIM	SR Testing Time
Dataset A [32]	3	33.28	0.9171	**0.275 s**
	4	**33.54**	**0.9190**	0.290 s
	5	33.50	0.9128	0.293 s
	6	33.44	0.9178	0.310 s
Dataset B [33]	3	31.72	0.8653	**0.266 s**
	4	**31.90**	**0.8677**	0.284 s
	5	31.67	0.8669	0.291 s
	6	31.62	0.8668	0.296 s

## Data Availability

No new data were created in this study. Data sharing is not applicable to this article.
